# A new lipoxygenase from the agaric fungus *Agrocybe aegerita*: Biochemical characterization and kinetic properties

**DOI:** 10.1371/journal.pone.0218625

**Published:** 2019-06-19

**Authors:** Dominik Karrer, Martin Rühl

**Affiliations:** 1 Institute of Food Chemistry and Food Biotechnology, Justus Liebig University Giessen, Giessen, Hesse, Germany; 2 Fraunhofer Institute for Molecular Biology and Applied Ecology IME Business Area Bioresources, Giessen, Hesse, Germany; Southern Illinois University School of Medicine, UNITED STATES

## Abstract

Oxylipins are metabolites with a variety of biological functions. However, the biosynthetic pathway is widely unknown. It is considered that the first step is the oxygenation of polyunsaturated fatty acids like linoleic acid. Therefore, a lipoxygenase (LOX) from the edible basidiomycete *Agrocybe aegerita* was investigated. The *Aae*LOX4 was heterologously expressed in *E*. *coli* and purified via affinity chromatography and gel filtration. Biochemical properties and kinetic parameters of the purified *Aae*LOX4 were determined with linoleic acid and linolenic acid as substrates. The obtained *K*_m_, *v*_max_ and *k*_cat_ values for linoleic acid were 295.5 μM, 16.5 μM · min^-1^ · mg^-1^ and 103.9 s^-1^, respectively. For linolenic acid *K*_m_, *v*_max_ and *k*_cat_ values of 634.2 μM, 19.5 μM · min^-1^ · mg^-1^ and 18.3 s^-1^ were calculated. Maximum activities were observed at pH 7.5 and 25 °C. The main product of linoleic acid conversion was identified with normal-phase HPLC. This analysis revealed an explicit production of 13-hydroperoxy-9,11-octadecadienoic acid (13-HPOD). The experimental regio specificity is underpinned by the amino acid residues W384, F450, R594 and V635 considered relevant for regio specificity in LOX. In conclusion, HPLC-analysis and alignments revealed that *Aae*LOX4 is a 13-LOX.

## Introduction

Oxylipins are lipoxygenase-derived metabolites with a variety of different structures and biological functions. These metabolites can be found in plants, mammals, bacteria, algae, mosses and fungi [1]. In mammals, they are involved in the regulation of immune response. In recent years, human lipid mediators like e.g. hepoxilins and trioxilins, which play an important role in tissue healing, organ protection, pain reduction and host defense received a lot of attention [2, 3].

In plants, they serve as signaling molecules, are involved in mediate stress response or in defense against pathogens, whereas in fungi they are associated with sporulation and the sexual as well as asexual life cycle [4, 5]. Lipoxygenases (LOX) are non-heme iron dependent dioxygenases which catalyze the insertion of molecular oxygen at a (1*Z*,4*Z*)-pentadiene motif, which occurs e.g. in polyunsaturated fatty acids (PUFAs), in a regio- and stereospecific manner [2, 3, 6, 7]. Additionally, fungal LOX with manganese instead of iron in the active site were also described [8, 9]. The oxidation of PUFAs leads to a reactive hydroperoxide and is considered to be the first step in the oxylipin-pathway. In higher fungi of the phyla Basidiomycota and Ascomycota, C_18_-PUFAs are predominantly present, whereas C_20_-PUFAs can be found only in very minor amounts [1]. This indicates that the oxygenation of linoleic-, linolenic- or arachidonic acid is the initial step in oxylipin-biosynthesis. Subsequent steps lead to a variety of oxylipins such as C8 volatile compounds which contribute to the significant flavor of mushroom. Among them, the most important and well known is oct-1-en-3-ol derived from linoleic acid [10]. The pathways, leading to oct-1-en-3-ol and other C8 volatile compounds are unknown. Several studies showed that fungi can convert linoleic acid into different oxylipins. It was found that the button mushroom *Agaricus bisporus* is able to produce oct-1-en-3-ol in several steps from linoleic acid. These steps include amongst others an oxygenation of linoleic acid to 10-hydroperoxyoctadecadienoic acid (10-HPOD). It was proposed that LOX generate the needed 10-HPOD, but up to now the corresponding enzyme yet has to be found [11, 12, 13]. Another study with *A*. *bisporus* revealed the conversion of linoleic acid into 8(*R*)-hydroxy-9*Z*,12*Z*-octadecadienoic acid and 8(*R*)-11(*S*)dihydroxy-9*Z*,12*Z*-octadecadienoic acid [14]. It also has been reported, that a mycelial pellet homogenate of an oyster mushroom *Pleurotus pulmonaris* produced oct-1-en-3-ol and the 10-oxo-acid from linoleic acid [15]. In general, studies on basidiomycetous LOX are scarce with only two LOX described so far [6, 10]. This shortcoming is addressed in this paper and a new LOX from the black poplar mushroom *Agrocybe aegerita*, also known to produce C8 volatile compounds [16, 17], is expressed, purified and characterized.

## Materials and methods

### Cloning and protein expression of *Aae*LOX4

The codon optimized *Aae*LOX4 gene (*Aaelox4*), whose original cDNA was deposited under the accession number MK451709, was commercially purchased and cloned into the plasmid pET28a (BioCat GmbH, Heidelberg, Germany). For protein expression, pET28a/*Aae*LOX4 plasmid was transformed into *E*. *coli* BL21(DE3) Gold. Recombinant *E*. *coli* cells were cultivated in Terrific Broth medium (TB) containing 12 g tryptone, 24 g yeast extract and 5 g glycerol, supplemented with 50 mg·L^-1^ kanamycin as selection marker, at 37 °C until an OD_600_ of 0.5 was reached. Expression was induced by adding isopropyl-β-d-thiogalactopyranoside to a final concentration of 0.5 mM. Expression temperature was reduced to 24 °C and cultivated for another 24 h. Cells were harvested by centrifugation (4,000 *g*, 30 min, 4 °C) and stored at -20 °C until further use.

### Protein purification

The cell pellet was thawed on ice and resuspended in lysis-buffer (50 mM phosphate, 300 mM NaCl, pH 7.5). Disruption of cells was carried out by sonification (3 cycles for 60 s on, 60 s rest) on ice using a sonifier (Bandelin Sonopuls, Berlin, Germany). After complete disruption, cell debris was removed by centrifugation (14,000 *g*, 30 min, 4 °C). The resulting supernatant was loaded twice on a 5 mL HisTrap column (GE-Healthcare), washed with 5 column volumes lysis-buffer containing 25 mM imidazole and eluted with 5 column volumes with the same buffer containing 500 mM imidazole. Gel filtration was performed on a Superdex 200 16/60 (GE Healthcare) using a buffer containing 50 mM Tris(hydroxymethyl)amino-methane (Tris/HCl), 300 mM NaCl, 10% (v/v) glycerol, pH 7.5 and a flow rate of 0.2 mL·min^-1^. Elution was collected in 2 mL fractions and analyzed via SDS-PAGE. Fractions with purified *Aae* -LOX4 were used for further analysis.

### Lipoxygenase assay

The substrate solution for the LOX assay was prepared as follows. 10 μL linoleic acid or linolenic acid was added to 915 μL ddH_2_O containing 15 μL Tween 20 and 60 μL 1M NaOH. Aliquots were stored at -20 °C and thawed before use. LOX activity was determined by recording the formation of the conjugated double bond at 234 nm (ε = 25,000 M^-1^·cm^-1^) on a Nanophotometer (Implen, Munich, Germany). Typical reaction mixture contained 0.5 mM linoleic acid, 20 μL enzyme solution and 50 mM phosphate buffer, pH 7.5 to a final volume of 1 mL.

### Determination of pH- and temperature-optimum

For the determination of the pH-optimum, three different buffers were used: 50 mM acetate buffer, pH 4.5–6; 50 mM phosphate buffer, pH 6.5–8 and 50 mM borate buffer, pH 8.5–10. Effects of the temperature were determined by incubating the reaction mixture at different temperatures, ranging from 15 °C– 65 °C.

### Kinetic parameters

Michaelis-Menten kinetics were calculated by incubating purified *Aae*LOX4 with different linoleic acid or linolenic acid concentrations ranging from 0.025 mM– 2 mM. All measurements were carried out in 50 mM phosphate buffer at pH 7.5 and 25 °C. Initial formation rates of the conjugated double bond were recorded at 234 nm in triplicates and consulted for calculating *K*_m_, *v*_max_ and *k*_cat_.

### Product analysis

Product specificity of *Aae*LOX4 was determined by incubating 1 mM linoleic acid, 20 μL *Aae*LOX4 and 50 mM phosphate buffer, pH 7.5 in a final volume of 1 mL. Reaction progress was followed by recording the formation of the conjugated double bond at 234 nm. Incubations were stopped when no further increase in extinction was detectable. The resulting hydroperoxy linoleic acids were extracted by adding 1 mL n-hexane. For HPLC-analysis, the extracts were dried under a continuous N_2_ stream and dissolved in n-hexane/2-propanol/acetic acid (100/5/1, v/v/v). Normal-phase-HPLC was carried out on a Nucleodur SiOH 100–5 column (Macherey-Nagel; 4.6x250 mm, 5 μm particle size) with an isocratic eluent system of n-hexane/2-propanol/acetic acid (100/5/1, v/v/v) at a flow rate of 1 mL·min^-1^. Elution was followed at 234 nm for hydroperoxy fatty acids and 210 nm for unsaturated fatty acids. Product specificity was evaluated by comparison with 13-HPOD and 9-HPOD standards prepared with commercial soybean LOX-1 [10]. Control experiments were carried out like described above without adding enzyme to the reaction mixture.

## Results

### Lipoxygenases of the black poplar mushroom *Agrocybe aegerita*

In the recently sequenced genome of *Agrocybe aegerita* (also known as *Cyclocybe aegerita* and *Pholiota aegerita*), five genes coding for putative LOX have been annotated and labelled as LOX1 (AAE3_00896), LOX2 (AAE3_01552), LOX3 (AAE3_09652), LOX4 (AAE3_04864) and LOX5 (AAE3_07753) ([18], www.thines-lab.senckenberg.de/agrocybe_genome). Deduced amino acid sequences of *A*. *aegerita* LOX genes and already characterized fungal LOX from Ascomycota (*Fusarium oxysporum* and *Gaeumannomyces graminis*) and Basidiomycota (*Pleurotus sapidus* and *Pleurotus ostreatus*) were used for phylogenetic analysis (http://www.phylogeny.fr/ using default parameters). LOX separated into two distinct sections with the characterized LOX from *G*. *graminis* [19] on one side and all basidiomycetous LOX as well as the characterized LOX from *F*. *oxysporum* [20] on the other side (S1 Fig). The latter group split into three parts: i) a large cluster containing both characterized LOXs from the *Pleurotus* species and four putative LOXs from *A*. *aegerita* (LOX1, LOX2, LOX4, LOX5), ii) LOX3 from *A*. *aegerita* and iii) the LOX from *F*. *oxysporum*.

### Heterologous expression and purification

**Fig 1:**
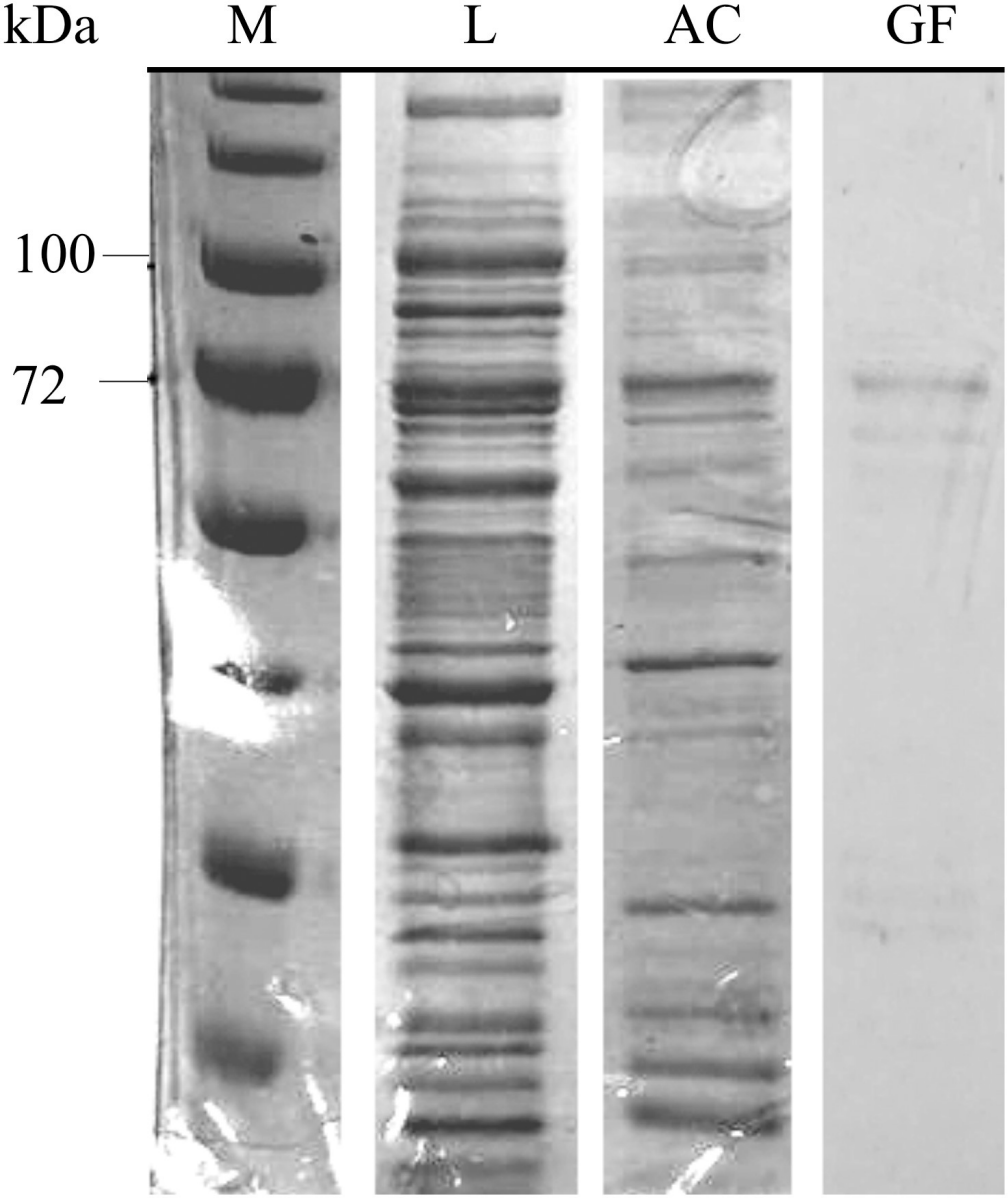
SDS-PAGE analysis of *Aae*LOX4 expression and purification. M = marker, L = cleared lysate, AC = Purification after affinity chromatography, GF = Purified enzyme after gel filtration.

Active and soluble *Aae*LOX4 was obtained from cultures of *E*. *coli* BL21(DE3) Gold carrying the pET28a/*Aae*LOX4 plasmid. SDS-PAGE analysis of cell lysate indicated that *Aae*LOX4 is present in the soluble lysate fraction (Fig 1). Thus, the cell lysate was directly applied onto an affinity chromatographic column, which resulted in the removal of crude impurities. Subsequent gel filtration led to pure *Aae*LOX4 (Fig 1). The main band had an estimated molecular weight of around 80 kDa confirming the predicted molecular weight of the amino acid sequence of 79 kDa. Overall, a 1,351-fold purification yield was obtained (Table 1).

**Table 1:** Summary of the purification steps of *Aae*LOX4 in *E*. *coli*.

Step	Total protein (mg)	Specific activity (U · mg^-1^)	Purification (fold)
Crude extract	146.97	0.038	1.0
Affinity chromatography	37.16	0.329	8.6
Gel filtration	0.09	51.34	1351.1

### LOX biochemical properties and characteristics

**Fig 2:**
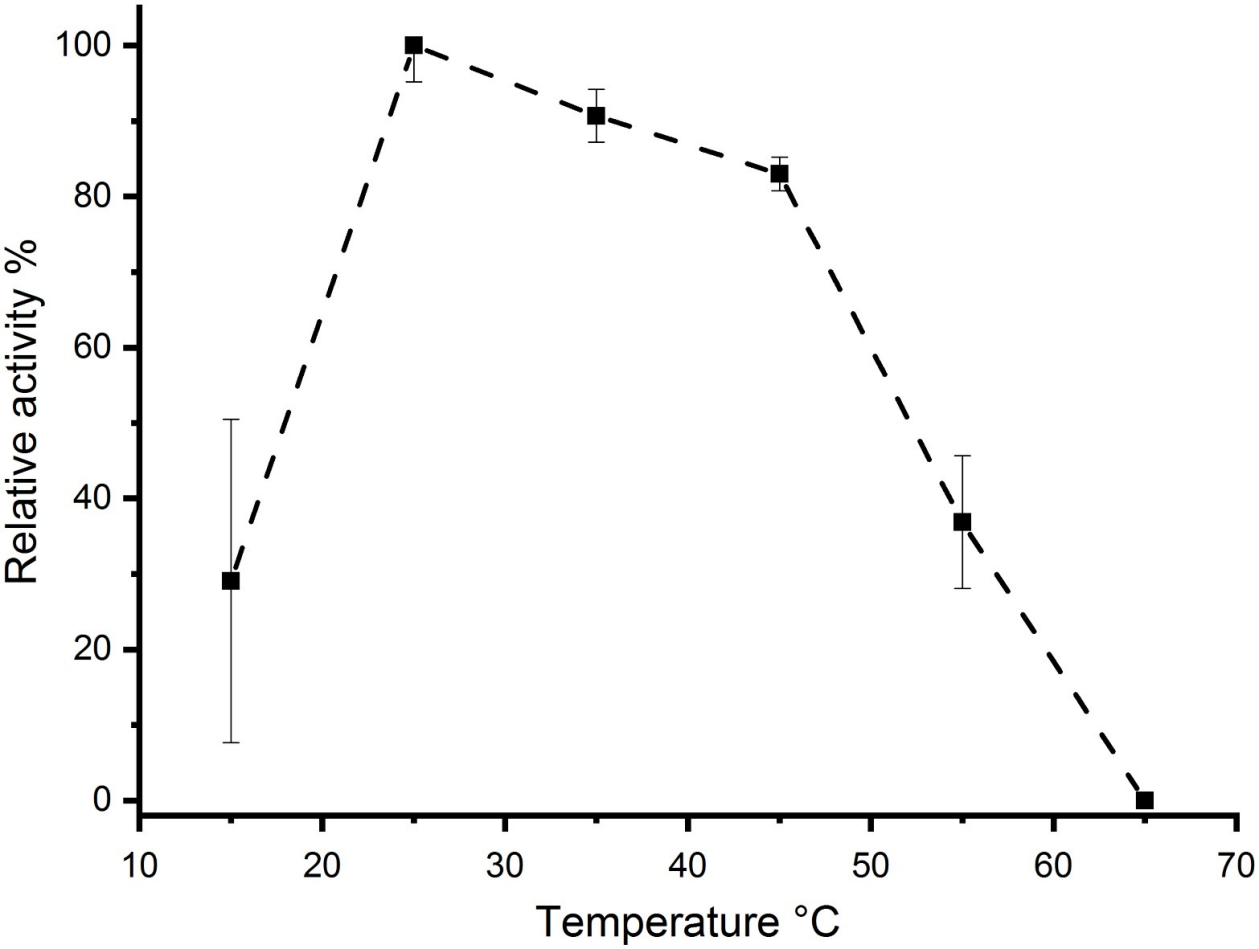
Effects of the temperature on *Aae*LOX4 activity.

The activity of *Aae*LOX4 was determined in a temperature range between 15 °C and 65 °C. While the relative activity from 25 °C to 45 °C resulted in a steady loss of about one-fifth of activity, a further temperature increase to 55 °C caused a dramatic loss in activity and no activity could be detected at 65 °C. At the lowest analyzed temperature of 15 °C, one-third of the maximal LOX activity remained (Fig 2).

**Fig 3:**
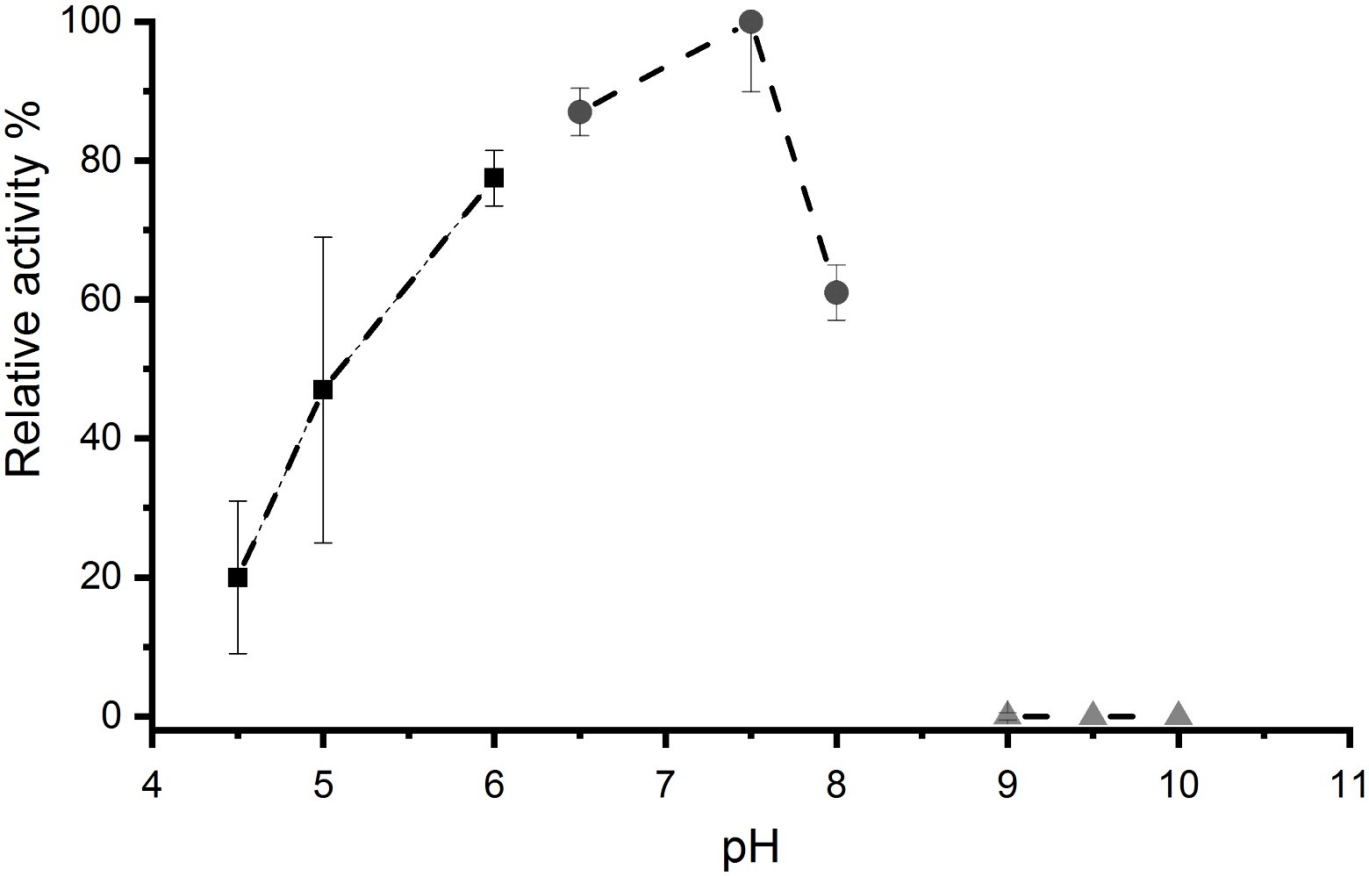
Effects of pH on *Aae*LOX4 activity. 50 mM acetate buffer (squares), 50 mM phosphate buffer (circles), 50 mM borate buffer (triangles).

With increasing pH of the buffer system used for LOX activity assays, a steady activity enhancement is obtained reaching its maximum at pH 7.5. Further increase of the pH showed a drastic activity loss with no detectable activity from pH 9 and above. In the acidic environment, *Aae*LOX4 showed more tolerable activity properties. Decreasing the pH from 7.5 to 6, resulted in a loss of about 20% activity. Nevertheless, at pH 5.0 the relative activity decreased to 50%, while 20% relative activity was left at pH 4.5 (Fig 3).

**Fig 4:**
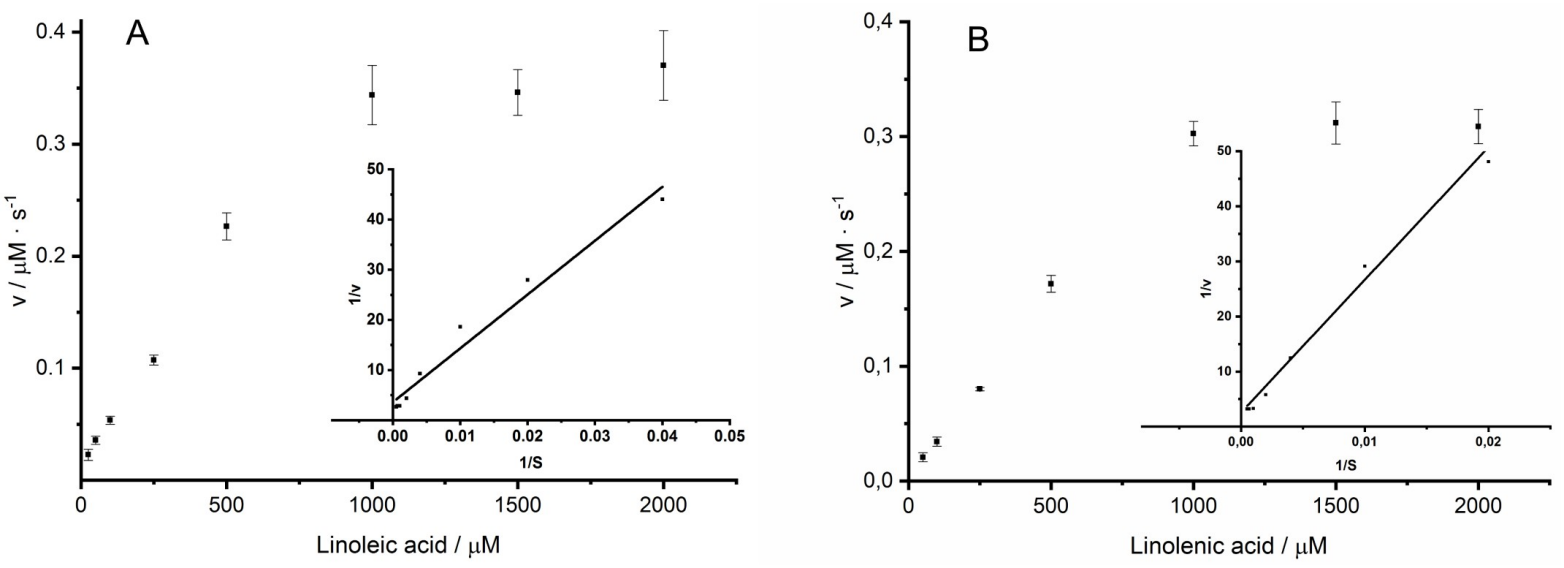
Velocity of *Aae*LOX4 dependent on linoleic acid (A) and linolenic acid (B) concentrations. Values represent the means of triplicates and their standard deviation. Inset represents Lineweaver-Burk plot.

Michaelis-Menten parameters were deduced on basis of the Lineweaver-Burk plot depicting *Aae*LOX4 activity with linoleic acid or linolenic acid concentrations between 0.025–2 mM (Fig 4). The obtained *K*_m_, *v*_max_ and *k*_cat_ values for linoleic acid were 295.5 μM, 16.5 μM min^-1^ mg^-1^ and 103.9 s^-1^, respectively. For linolenic acid as substrate *K*_m_, *v*_max_ and *k*_cat_ values of 634.2 μM, 19.5 μM · min^-1^ · mg^-1^ and 18.3 s^-1^ were calculated (Table 2). Substrate specificity of *Aae*LOX4 was estimated by comparing the relative activities towards different PUFAs (Table 3). The enzyme showed the highest specificity for linoleic acid and a high relative activity for linolenic acid (88.8%). However, arachidonic acid showed the lowest relative activity with 7.3%.

**Table 2:** Kinetic parameters of *Aae*LOX4.

Substrate	*K*_m_ / μM	*v*_max_ / μM · min^-1^ · mg^-1^	*k*_cat_ / s^-1^
linoleic acid	295.5	16.5	103.9
linolenic acid	634.2	19.5	18.3

**Table 3:** Substrate specificity of *Aae*LOX4.

Substrate	Relative activity / %
linoleic acid	100
linolenic acid	88.8
arachidonic acid	7.3

### HPLC-analysis of products

**Fig 5:**
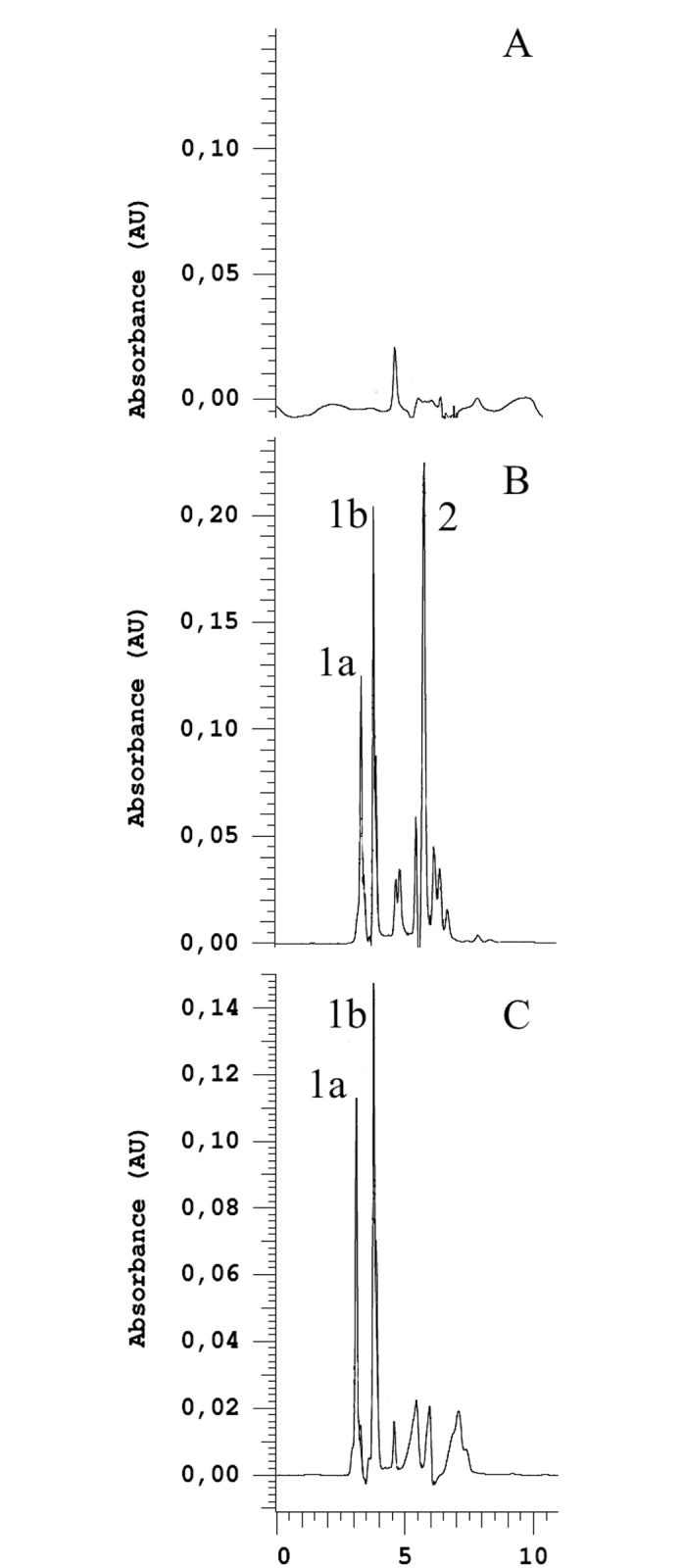
NP-HPLC analysis of the oxygenated products of linoleic acid. Chromatogram was recorded at 234 nm. (A) negative control. Extract of the reaction mixture lacking enzyme. (B) Extract of the reaction products from soybean LOX-1. (C) Extract of the reaction products from *Aae*LOX4. (1a) 13-*Z*,*E*-HPOD, (1b) 13-*E*,*E*-HPOD, (2) 9-*Z*,*E*-HPOD + 9-*E*,*E*-HPOD.

Specificity of *Aae*LOX4 was analyzed by normal-phase HPLC. The elution profile of *Aae*LOX4 shows two major products which elute at 3.12 min and 3.81 min (Fig 5). 13-*Z*,*E*-HPOD and 13-*E*,*E*-HPOD as well as 9-*Z*,*E*-HPOD and 9-*E*,*E*-HPOD have been prepared with soybean LOX-1 according to Kuribayashi et al. [10]. 13-*Z*,*E*-HPOD and 13-*E*,*E*-HPOD elute at 3.33 min and 3.79 min, respectively, whereas the isomeric 9-HPOD mixture elute as one peak at 5.79 min. The negative control showed no peaks at these retention times. The comparison between *Aae*LOX4 products and soybean LOX-1 products revealed that *Aae*LOX4 produces highly specific 13-HPOD. A production of 9-HPOD by *Aae*LOX4 was not detectable and ruled out by comparing the UV-spectra of 9-HPOD eluting at 5.79 min from soybean LOX-1 with peaks in this range in the elution profile of *Aae*LOX4 products.

## Discussion

In this study, we were able to express a soluble and active *Aae*LOX4 from *A*. *aegerita* in *E*. *coli*. As far as we know, this is the second basidiomycetous LOX recombinantly expressed in *E*. *coli*. While Plagemann et al. [5] used an approach with co-expression of chaperones to accomplish a soluble LOX expression from cDNA of *P*. *sapidus*, we reached the soluble expression with a codon optimized gene. Affinity chromatography with following gel filtration led to a highly purified protein in sufficient concentrations for further characterization experiments. The observed influence of temperature and pH on *Aae*LOX4 with its maximum activity at 25 °C and pH 7.5 is in accordance with LOXs from other fungi, such as a wildtype LOX from *P*. *ostreatus*, having its maximum activity at 25 °C and pH values between 7.5 and 8.0 [10]. However, the recombinant LOX from *P*. *sapidus* showed a temperature range with highest activities ranging from 25 °C to 35 °C. The optimal pH value of *P*. *sapidus* LOX was 7.0 and in contrast to *Aae*LOX4 showed activity up to a pH of 9.5 [5]. Interestingly, *Aae*LOX4 showed differences in the kinetic parameters for linoleic acid compared to other LOXs from Basidiomycota. The *K*_m_ values for the LOXs from *P*. *sapidus* and from *P*. *ostreatus* are 40.3 μM and 130 μM, which are both lower than our calculated *K*_m_ of 295.5 μM. On the other hand, the obtained *v*_max_ of 16.5 μM min^-1^ mg^-1^ is comparable with the LOX from *P*. *ostreatus* (23.4 μM min^-1^ mg^-1^) and the *k*_cat_ value of 103.9 s^-1^ is comparable with the LOX from *P*. *sapidus* (157 s^-1^) [5, 10]. Analogous *K*_m_ and *v*_max_ values were also found for a LOX in oriental melon (230.3 μM and 15.25 μM min^-1^ mg^-1^) [21]. Interestingly, a LOX originating the bacteria *Myxococcus xanthus* shows comparable *K*_m_ values for linoleic acid (380 μM) but drastically lower *K*_m_ with linolenic acid and other C_20_-PUFAs like arachidonic acid or eicosapentaenoic acid [6]. Soybean LOX-1 shows a lower *K*_m_ of 150 μM but shares a comparable *v*_max_ of 13.5 μM min^-1^ mg^-1^ [22]. The catalytic efficiency of *Aae*LOX4 was higher with linoleic acid than with linolenic acid. This finding reflects the estimated relative activities towards different PUFAs which revealed a preference for linoleic acid. High activity was also found for linolenic acid, whereas arachidonic acid showed poor activity. These results are comparable with the LOXs from *P*. *ostreatus* and *F*. *oxysporum* [10, 20]. The analysis of product specificity revealed that 13-HPOD (13-*Z*,*E*-HPOD and 13-*E*,*E*-HPOD) was the only detectable product and thus, *Aae*LOX4 can be considered as a 13-LOX. Equally, LOXs from *P*. *sapidus*, *P*. *ostreatus*, *F*. *oxysporum* and *G*. *graminis* showed a specific conversion of linoleic acid to 13-HPOD [5, 10, 19, 20]. Nevertheless in contrast to *Aae*LOX4, LOXs from *P*. *sapidus*, *P*. *ostreatus* and *F*. *oxysporum* produced minor amounts of 9-HPOD. Whereas the LOX from *G*. *graminis* showed no significant synthesis of 9-HPOD [19]. This finding was confirmed with amino acid sequence alignments which showed that various determinants responsible for the regio- and stereo-specificity can also be found in *Aae*LOX4 (S2 Fig). Three positions, which are responsible for the depth of substrate penetration and thus are responsible for regio-specificity, have been described for mammalian LOX [23, 24]. While space-filling amino acids at these positions lead to oxygenation near the methyl end, enlargement of the active site favors oxygenations towards the carboxy end [24]. Applying this concept on *Aae*LOX4, the bulky amino acids at the crucial positions (W384, F450 and V635) are present in the amino acid sequence of *Aae*LOX4 and result in the transformation of linoleic acid to 13-HPOD (Fig 5). Also the LOXs from *P*. *ostreatus*, *P*. *sapidus* and *F*. *oxysporum* led to a specific production of 13-HPOD [5, 10, 20]. Consistently, all these LOXs share the same bulky amino acids at the crucial positions [23, 24]. According to Andreou et al. [25, 26] and Hornung et al. [27], regio-specificity is mainly determined by the orientation of the substrate. Based on their model, substrate orientation with the methyl end first leads to an oxygenation at C-13. The invers orientation of the substrate therefore is proposed to oxygenate C-9. Additionally, reduced space in the active site is also considered to favor a substrate orientation with its methyl end towards the active site. This model involves the importance of a positive charged amino acid at the end of the active site with no interference from another amino acid masking the positive charge to stabilize the carboxy end of the substrate. On the one hand the LOXs from *P*. *ostreatus*, *P*. *sapidus* and *F*. *oxysporum* share a lysine, considered to stabilize the negative charged carboxy end of the substrate and therefore differ in their amino acid sequence compared to *Aae*LOX4 where this lysine is replaced by an arginine which could of course fulfil the same purpose. On the other hand, the alignment shows that the LOXs from *P*. *ostreatus*, *P*. *sapidus*, *F*. *oxysporum* and *Aae*LOX4 share a phenylalanine near the positive charged amino acids lysine or arginine which masks the charge and therefore leads to a substrate orientation with the methyl end penetrating the active site leading to a region-specific oxygenation at C-13 [25, 26, 27]. Phylogenetic analysis shows that the fungal LOXs cluster in different groups. Whether LOXs of the same group harbor similar enzymatic properties is an important question, which can help to depict distinct enzyme function. This assumption is reasonable by comparing the *K*_m_ values for linoleic acid LOXs in the phylogenetic tree. *Fox*LOX and *Ggr*LOX, show low *K*_m_ values of 4.4 and 6.9 μM while the subgroup of *Pos*LOX and *Psa*LOX show distinctly higher *K*_m_ values of 130 and 40.3 μM, respectively. The characterized *Aae*LOX4, which does not cluster with other subgroups of characterized LOX showed an even higher *K*_m_ value of 295.5.

## Supporting information

S1 Fig Phylogenetic analysis of different fungal LOX. *Aae—Agrocybe aegerita*, *Fox—Fusarium oxysporum*, *Ggr—Gaeumannomyces graminis*, *Pos—Pleurotus ostreatus*, *Psa—Pleurotus sapidus*; *Aae*LOX1, *Aae*LOX2, *Aae*LOX3, *Aae*LOX4 (MK451709), *Aae*LOX5, *Fox*LOX (KNB01601), *Ggr*LOX (AAK81882), *Psa*LOX (CCV01580), *Pos*LOX (CCV01578).
(TIF)

S2 Fig Partial amino acid sequence alignment of different LOX. *Agrocybe aegerita Aae*LOX1, *Aae*LOX2, *Aae*LOX3, *Aae*LOX4, *Aae*LOX5, *Pleurotus sapidus Psa*LOX, *Pleurotus ostreatus Pos*LOX, *Fusarium oxysporum Fox*LOX, *Gaeumannomyces graminis Ggr*LOX. Alignment was carried out by using Clustal Omega with default parameters. The highlighted amino acid residues involved in ligand binding are indicated as “L”. Stereo specificity related amino acids are indicated as “B” [24], “H” [27] and “S” [23].
(TIF)

## References

[1] Brodhun F, Feussner I. Oxylipins in fungi. FEBS J. 2011;278(7):1047–1063. 10.1111/j.1742-4658.2011.08027.x 21281447

[2] An JU, Song YS, Kim KR, Ki YJ, Yoon DY, Oh DK. Biotransformation of polyunsaturated fatty acids to bioactive hepoxilins and trioxilins by microbial enzymes. Nat Commun. 2018;9(1):128 10.1038/s41467-017-02543-8 29317615 PMC5760719

[3] Lee IG, An JU, Ko YJ, Park JB, Oh DK. Enzymatic synthesis of new hepoxilins and trioxilins from polyunsaturated fatty acids. Green Chem. 2019 10.1039/c9gc01031a

[4] Kock JLF, Venter P, Linke D, Schewe T, Nigam S. Biological dynamics and distribution of 3-hydroxy fatty acids in the yeast *Dipodascopsis uninucleata* as investigated by immunofluorescence microscopy. Evidence for a putative regulatory role in the sexual reproductive cycle 1. FEBS Lett. 1998;427(3):345–348. 9637254 10.1016/s0014-5793(98)00406-2

[5] Plagemann I, Zelena K, Arendt P, Ringel PD, Krings U, Berger R. LOXPsa1, the first recombinant lipoxygenase from a basidiomycete fungus. J Mol Catal B: Enzym. 2013;87(1):99–104.

[6] An JU, Hong SH, Oh DK, Regiospecificity of a novel bacterial lipoxygenase from *Myxococcus xanthus* for polyunsaturated fatty acids. Biochim Biophys Acta, Mol Cell Biol Lipids. 2018;8(8):823–833. 10.1016/j.bbalip.2018.04.014 29684557

[7] Liavonchanka A, Feussner I. Lipoxygenases: occurrence, functions and catalysis. J Plant Physiol. 2006;163(3):348–357. 10.1016/j.jplph.2005.11.006 16386332

[8] Hamberg M, Su C, Oliw E. Manganese Lipoxygenase. Discovery of a bis-allylic hydroperoxide as product and intermediate in a lipoxygenase reaction. J Biol Chem. 1998;273(21):13080–13088. 10.1074/jbc.273.21.13080 9582346

[9] Oliw EH, Jernerén F, Hoffmann I, Sahlin M, Garscha U. Manganese lipoxygenase oxidizes bis-allylic hydroperoxides and octadecenoic acids by different mechanisms. Biochim Biophys Acta. 2011;1811(3):138–147. 10.1016/j.bbalip.2010.12.002 21167311

[10] Kuribayashi T, Kaise H, Uno C, Hara T, Hayakawa T, Joh T. Purification and Characterization of Lipoxygenase from *Pleurotus ostreatus*. J Agric Food Chem. 2002;50(5):1247–1253. 11853512 10.1021/jf0112217

[11] Wurzenberger M, Grosch W. The enzymic oxidative breakdown of linoleic acid in mushrooms (*Psalliota bispora*). Z Lebensm Unters Forsch. 1982;175(3):186–190.

[12] Wurzenberger M, Grosch W. Origin of the oxygen in the products of the enzymatic cleavage reaction of linoleic acid to 1-octen-3-ol and 10-oxo-trans-8-decenoic acid in mushrooms (*Psalliota bispora*). Biochim Biophys Acta. 1984;794(1):18–24.

[13] Wurzenberger M, Grosch W. Stereochemistry of the cleavage of the 10-hydroperoxide isomer of linoleic acid to 1-octen-3-ol by a hydroperoxide lyase from mushrooms (*Psalliota bispora*). Biochim Biophys Acta. 1984;795(1):163–165.

[14] Wadman M, van Zadelhoff G, Hamberg M, Visser T, Veldink G, Vliegenthart JG. Conversion of linoleic acid into novel oxylipins by the mushroom *Agaricus bisporus*. Lipids. 2005;40(11):1163–1170. 16459929 10.1007/s11745-005-1481-2

[15] Assaf S, Hadar Y, Dosoretz C. Biosynthesis of 13-hydroperoxylinoleate, 10-oxo-8-decenoic acid and 1-octen-3-ol from linoleic acid by a mycelial-pellet homogenate of *Pleurotus pulmonarius*. J Agric Food Chem. 1995;43(8):2173–2178.

[16] Rapior S, Breheret S, Talou T, Pélissier Y, Milhau M, Bessière JM. Volatile Components of Fresh *Agrocybe aegerita* and *Tricholoma sulfureum*. Cryptogam Mycol. 1998;19(1):15–23.

[17] Kleofas V, Sommer L, Fraatz MA, Zorn H, Rühl M. Fruiting body production and aroma profile analysis of *Agrocybe aegerita* cultivated on different substrates. Nat Resour. 2014;5(6):233–240.

[18] Gupta DK, Rühl M, Mishra B, Kleofas V, Hofrichter M, Herzog R, et al The genome sequence of the commercially cultivated mushroom *Agrocybe aegerita* reveals a conserved repertoire of fruiting-related genes and a versatile suite of biopolymer-degrading enzymes. BMC Genomics. 2018;19(1):48 10.1186/s12864-017-4430-y 29334897 PMC5769442

[19] Su C, Oliw E. Manganese Lipoxygenase. Purification and Characterization. J Biol Chem. 1998;273(21):13072–13079. 10.1074/jbc.273.21.13072 9582345

[20] Brodhun F, Cristobal-Sarramian A, Zabel S, Newie J, Hamberg M, Feussner I. An 13*S*-Lipoxygenase with an α-Linolenic Acid Specific Hydroperoxidase Activity from *Fusarium oxysporum*. PLoS ONE. 2013;8(5):5. 10.1371/journal.pone.0064919 PMC3669278 23741422

[21] Cao S, Chen H, Zhang C, Tang Y, Liu J, Qi H. Heterologous Expression and Biochemical Characterization of Two Lipoxygenases in Oriental Melon, *Cucumis melo var*. *makuwa Makino*. PLoS ONE. 2016;11(4):4. 10.1371/journal.pone.0153801 PMC4839669 27101009

[22] Pinto M, García-Barrado J, Macías P. Oxidation of resveratrol catalyzed by soybean lipoxygenase. J Agric Food Chem. 2003;51(6):1653–1657. 10.1021/jf025818d 12617600

[23] Sloane D, Leung R, Craik C, Sigal E. A primary determinant for lipoxygenase positional specificity. Nature. 1991;354(6349):149–152. 10.1038/354149a0 1944593

[24] Borngräber S, Browner M, Gillmor S, Gerth C, Anton M, Fletterick R, et al Shape and Specificity in Mammalian 15-Lipoxygenase Active Site. J Biol Chem. 1999;274(52):37345–37350. 10.1074/jbc.274.52.37345 10601303

[25] Andreou A, Feussner I. Lipoxygenases—Structure and reaction mechanism. Phytochemistry. 2009;70(13–14):1504–1510. 10.1016/j.phytochem.2009.05.008 19767040

[26] Andreou A, Hornung E, Kunze S, Rosahl S, Feussner I. On the substrate binding of linoleate 9-lipoxygenases. Lipids. 2009;44(3):207–215. 10.1007/s11745-008-3264-4 19037675

[27] Hornung E, Kunze S, Liavonchanka A, Zimmermann G, Kühn D, Fritsche K. Identification of an amino acid determinant of pH regiospecificity in a seed lipoxygenase from *Momordica charantia*. Phytochemistry. 2008;69(16):2774–2780. 10.1016/j.phytochem.2008.09.006 18945457

