# Supplementary figures and images for: A new lipoxygenase from the agaric fungus Agrocybe aegerita: Biochemical characterization and kinetic properties
Source: PLoS One. 2019 Jun 19;14(6):e0218625. doi: 10.1371/journal.pone.0218625 (PMC6584016; doi:10.1371/journal.pone.0218625)

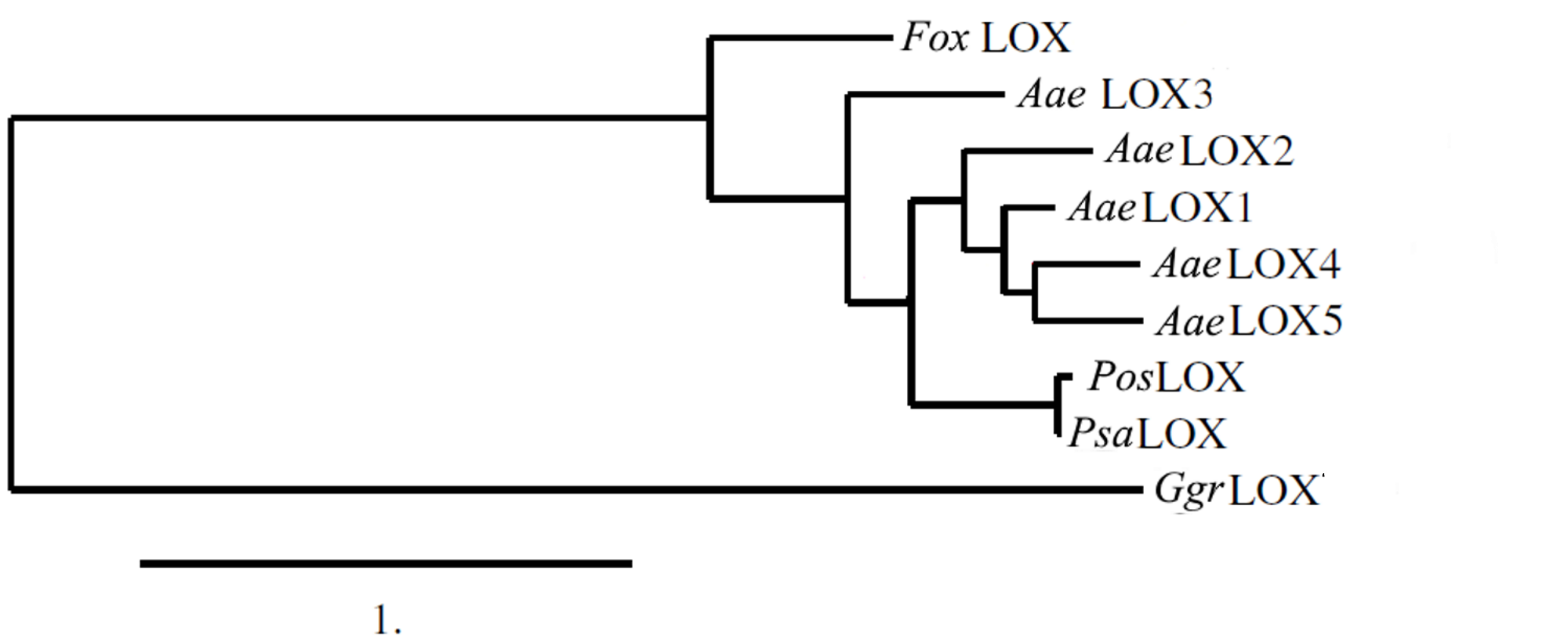

Supplement: S1 Fig — Aae—Agrocybe aegerita, Fox—Fusarium oxysporum, Ggr—Gaeumannomyces graminis, Pos—Pleurotus ostreatus, Psa—Pleurotus sapidus; AaeLOX1, AaeLOX2, AaeLOX3, AaeLOX4 (MK451709), AaeLOX5, FoxLOX (KNB01601), GgrLOX (AAK81882), PsaLOX (CCV01580), PosLOX (CCV01578). (TIF) [file pone.0218625.s001.tif]

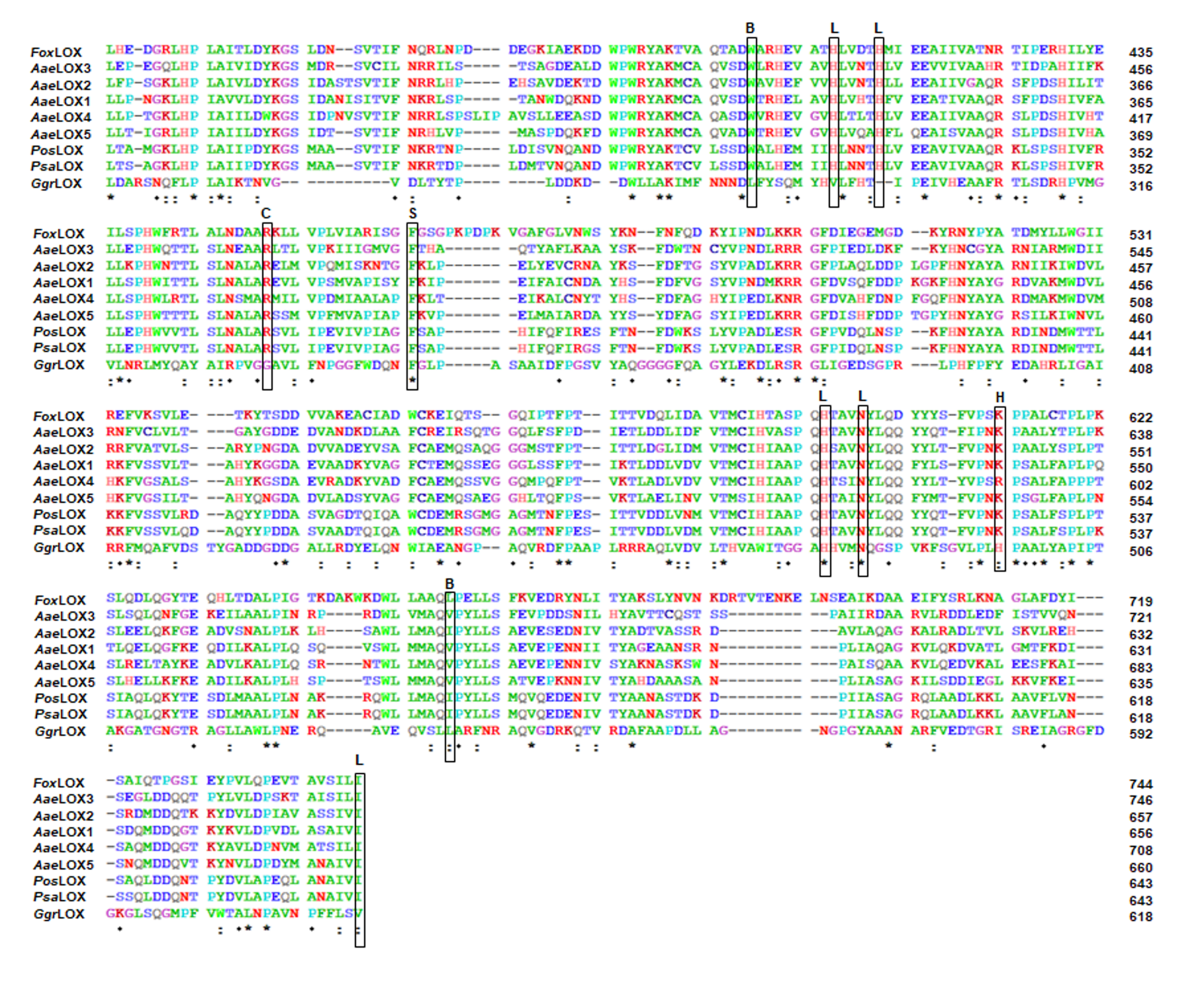

Supplement: S2 Fig — Agrocybe aegerita AaeLOX1, AaeLOX2, AaeLOX3, AaeLOX4, AaeLOX5, Pleurotus sapidus PsaLOX, Pleurotus ostreatus PosLOX, Fusarium oxysporum FoxLOX, Gaeumannomyces graminis GgrLOX. Alignment was carried out by using Clustal Omega with default parameters. The highlighted amino acid residues involved in ligand binding are indicated as “L”. Stereo specificity related amino acids are indicated as “B” [24], “H” [27] and “S” [23]. (TIF) [file pone.0218625.s002.tif]
